# Exosomes derived from HUVECs alleviate ischemia-reperfusion induced inflammation in neural cells by upregulating KLF14 expression

**DOI:** 10.3389/fphar.2024.1365928

**Published:** 2024-05-02

**Authors:** Jianxin Qin, Lihong Zhou, Lei Yu, Jingwen Ye, Feng Wang, Jin Zhou, Yunjuan Gu, Gang Chen, Xia Chen

**Affiliations:** ^1^ Department of Histology and Embryology, Medical School of Nantong University, Nantong, China; ^2^ Department of Endocrinology, Affiliated Hospital of Nantong University, Nantong University, Nantong, China; ^3^ Nantong Xingzhong Cell Engineering Co. Ltd, Nantong, China

**Keywords:** HUVEC, exosomes, brain ischemic, KLF14, inflammation

## Abstract

Neuroinflammation plays a key role in the progression of secondary brain injury after ischemic stroke, and exosomes have been increasingly recognized to eliminate inflammatory responses through various mechanisms. This study aimed to explore the effect and possible mechanism of human umbilical vein endothelial cells derived exosomes (H-EXOs) on neuroinflammation. We established a transient middle cerebral artery occlusion/reperfusion (tMCAO/R) in male rats and oxygen-glucose-deprivation/reoxygenation (OGD/R) model in cultured neurons to mimic secondary brain injury after ischemic stroke *in vivo*. H-EXOs were administered at the same time of reperfusion. Results showed that the production of pro-inflammatory cytokines TNF-α, IL-1β, and IL-6, and the transcription factor Krüppel-like factor 14 (KLF14) were significantly increased both in rat brain tissue and cultured neural cells after ischemic-reperfusion (I/R) injury. H-EXOs treatment significantly improved the cultured cell viability, reduced infarct sizes, mitigated neurobehavioral defects, and alleviated the expression of pro-inflammatory cytokines compared with the control group, indicating that H-EXOs exerted anti-inflammatory effect against I/R injury. Further studies revealed that the anti-inflammatory effect of H-EXOs could be weakened by small-interfering RNA (siKLF4) transfection. KLF14 was a protective factor produced during cerebral ischemia-reperfusion injury. In conclusion, H-EXOs protect neurons from inflammation after I/R injury by enhancing KLF14 expression.

## Introduction

Ischemic stroke is often caused by vascular blockage. The most common treatments for ischemic injuries rely on the immediate restoration of blood flow which is limited by the narrow time frame and invasiveness of the surgical procedures (Prabhakaran et al., 2015). Additionally, the subsequent reperfusion of the blood flow can lead to a secondary injury, termed as reperfusion injury, mainly owing to a series of pathological changes induced by ischemia-reperfusion, including blood-brain barrier (BBB) disruption, increased excitotoxicity, oxidative stress, inflammation, and mitochondrial dysfunction (Parvez et al., 2022). Of these events, the inflammatory reaction occurs initially in the acute phase and continues to escalate during the course of the injury. The main effects of inflammation are the destruction of the BBB, damage to neurons, and acceleration of vascular aging, all of which can exacerbate brain tissue damage and are related to the severity and prognosis of stroke (Kunz et al., 2010; Yang et al., 2021). In the clinical setting, the current treatment strategy for ischemic stroke relies on thrombolysis. However, thrombolytic therapy itself exacerbates the occurrence of inflammation and may cause greater damage to patients with ischemic stroke. Thus, the inhibition of inflammation is of great significance for the treatment of ischemic stroke (Wong and Crack, 2008; Shi et al., 2021).

Neuroinflammation participates in the whole pathophysiological process of cerebral ischemia (Kelly et al., 2018). Numerous studies have shown that stem cells can reduce delayed neuronal degeneration by inhibiting inflammatory responses. The neural factors secreted by stem cells can also regulate the inflammatory response after an infarction. Using stem cell transplantation to treat cerebral infarction may promote endogenous nerve regeneration and the self-repair of brain tissue. But stem cells have heterogeneity, which directly affects the therapeutic effect. Furthermore, some stem cell therapies have safety risks, mainly including tumorigenicity, abnormal immune response, and unexpected differentiation. Compared to stem cells, exosome-based cell-free therapies are gaining more attention because they effectively avoid the risks of low survival, strong immune rejection, and high mutational tumorigenicity associated with direct stem cell therapy (Verweij et al., 2019). Exosome is an extracellular vesicle with a diameter of about 40–140 nm that transmits biological information between cells, owing to its special structure and contents (including cell surface proteins and various components within cells such as DNA, RNA, lipids, metabolites, etc.) (Ferguson et al., 2018; Kalluri and LeBleu, 2020). Given their excellent biological characteristics, including biocompatibility, stability, low toxicity, low immunogenicity, and efficient exchange of molecular cargos, exosomes can be potentially applied for disease treatment (Patel et al., 2021; Wan et al., 2022; Xie et al., 2022). Recent studies demonstrated that exosomes derived from berberine-pretreated astrocytes, bone marrow-derived mesenchymal stem cells, endothelial progenitor cells, and neural stem cells can suppress neuroinflammation after stroke through different mechanisms (Liu et al., 2021; Ding et al., 2023; Pan et al., 2023; Peng et al., 2023). Studies suggested that exosomes have protective effects on the brain by inhibiting inflammatory responses following ischemic stroke and have broad application prospects.

The exosomes employed herein were derived from human umbilical vein endothelial cells (HUVECs), which are primary cells isolated from umbilical veins. HUVECs offer several advantages, such as easy isolation, a sufficient source, low immunogenicity, strong proliferative ability, and no ethical disputes. The blood in umbilical venous is usually arterial blood, which contains a certain amount of oxygen and nutrients (Yamaguchi et al., 1996). In addition, HUVECs synthesize and secrete various cytokines and chemicals which can regulate vasodilation and contraction, platelet aggregation, coagulation, etc. (Yamaguchi et al., 1998; Yildirim et al., 2005; Li et al., 2006). Overall, it indicates that exosomes derived from HUVEC (simply called H-EXOs) can be potential employed for ischemic stroke treatment, but more research are needed to strengthen that. Herein, we aim to explore the effect and underlying mechanism of H-EXOs on inflammation after reperfusion in cerebral ischemic injury.

In the present study, we performed transient right middle cerebral artery occlusion/reperfusion (tMCAO/R) model in rats and oxygen-glucose deprivation/re-oxygenation (OGD/R) model in cultured neural cells to simulate brain injury with I/R. We found that H-EXOs significantly protected the brain from ischemic damage by suppressing the production of pro-inflammatory factors and the phosphorylation of the P65 subunit of the nuclear factor kappa B (NF-κB). We also found that the H-EXOs have anti-inflammatory effects, at least in part, via upregulating the transcription factor Krüppel-like factor 14 (KLF14). Together, our findings provide a new experimental evidence of the pathophysiological mechanism underlying ischemic stroke, and highlight new potential targets for the prevention and treatment of ischemic brain injury.

## Materials and methods

The materials and reagents used in the experiment are detailed in Supplementary Table S1.

1. Cell Culture: Mouse hippocampal neuronal HT22 cell line and human umbilical vein endothelial cells (HUVEC) were cultured in Dulbecco’s modified Eagle’s medium (DMEM) supplemented with 10% fetal bovine serum (FBS) and 1% Penicillin-Streptomycin at 37 °C in a 5% CO_2_ incubator. The culture medium was changed daily to maintain cell viability.
2. Extraction and Identification of Exosomes: The exosomes were isolated through size exclusion chromatography (SEC) and subsequently gathered via ultracentrifugation. In brief, a chromatography column filled with porous gel particles was used for size-exclusion chromatography. Samples passed through the column, and substances larger than the gel particle pore size were unable to enter the pores and were washed out through the gaps between the gel particles. Substances smaller than the gel particle pore size entered the interior pores. In exosomes isolation, the larger-sized exosomes were eluted first, while smaller-sized free proteins and impurities took longer to elute, effectively separating them from the exosomes, then the pellets were collected by ultracentrifugation at 120,000 g for 90 min.
3. Exosome fluorescence labeling and internalization experiments: The exosomes were labeled using the PKH26 Red Fluorescent Probe as per the provided instructions (Liu et al., 2023). Subsequent tail vein injections in MCAO/R-injured rats were performed using fluorescently labeled exosomes. Brain sections were taken 4 h later. HT22 neuronal cells were cultured on circular slides. Then, luorescently labeled exosomes were added to the well for 12 h. Make a glass coverslip mounted on a glass microscope slide. Fluorescent images were taken using confocal microscopy.
4. Establishment of the OGD/R injured model: The OGD/R injured model was created using the methods previously described with some modifications (Wang et al., 2018). HT22 cells were cultured with 1 mM Na2S2O4 (oxygen scavenger) in sugar-free DMEM for 4 h, then subjected reperfusion for 12 h to construct a stable OGD/R-injured model.
5. Detection of cell viability: 3 - (4,5 - dimethylthiazol - 2 - yl) −2,5 - diphenyl - 2H - tetrazolium bromide (MTT) test was used to assess cell viability. The cultured cells were cultured in a 96-well plate with a density of 4×10^3^ cells per well, then exposed to OGD/R treatment and other relevant protocol. 10 μL of 0.5% MTT solution prepared from MTT powder was added to each well and incubated for 4 h in a CO_2_ incubator. Next, a 100 μL solution of 10% SDS, which was made from SDS powder, was introduced into each well of the culture. This process was allowed to proceed for a duration of 12 h, following which the absorbance at 570 nm was determined using an enzyme meter.
6. Cell Transfection: The transfection of siRNA was conducted by employing the Lipofectamine 2000 reagent in accordance with the guidelines provided by the manufacturer. KLF14 siRNA and negative controls were obtained from Ribobio. The sequences used for cell transfection are as follows: 5′- GCA​ACA​AGG​CCT​ACT​ACA​A -3′. KLF14 overexpression lentivirus was purchased from Genechem, and transfection was performed according to the manufacturer’s instructions.
7. MCAO/R Model: Adult male SD rats used in this study, weighted 250–280 g were obtained from the Model Animal Research Center of Nantong University and received ethical approval for animal experimentation (No. S20231219-001). Premade poly-L-lysine-coated sutures were purchased from Cionotech. In brief, rats were anesthetized with isoflurane, and after skin preparation, a minor surgical cut was performed along the centerline of the neck. The right common carotid artery was exposed, and a small cut was made in it. The suture was inserted through this cut, advanced into the internal carotid artery, and ultimately reached the middle cerebral artery, where it was secured using a pre-tied ligature. After 2 h of blockage, the rats were followed by reperfusion for 24 h. During the surgery, the rectal temperature of the rats was maintained at 36.5°C ± 0.5°C, and their respiration and heartbeat were monitored. Follow-up experiments were conducted using rats with Longa behavioral scores of 1–3 (Mahemuti et al., 2023).
8. Measurement of Cerebral Infarct Volume: 2, 3, 5-triphenyltetrazolium chloride (TTC) staining was performed 24 h after MCAO surgery. Brains from each group of rats were rapidly put on ice and sliced into approximately 2 mm-thick sections. These slices were then placed in a 2% TTC solution prepared with TTC powder at 37 °C for 30 min. Normal brain tissue appeared red, while ischemic brain tissue appeared white (Tang et al., 2024). The total area of the infarct in each section was calculated and presented as a percentage of the volume of the unaffected area: infarct volume (%) = [(volume of the healthy side hemisphere - volume of the uninjured area on the affected side)/volume of the healthy side hemisphere] × 100%.
9. Longa Score: 24 h after completion of the MCAO model, the examiner used the Longa behavioral test to evaluate the neurological impairment score (Longa et al., 1989). The Longa Score was evaluated using a scale ranging 0–5. A score of 0 indicated the absence of any neurological deficits. A score of 1 indicated the inability to fully extend the left forepaw. Circling to the left when walking was assigned a score of 2. Falling to the left while walking corresponded to a score of 3. A score of 4 indicated the inability to walk spontaneously and a lack of consciousness. Higher scores on this scale were indicative of more severe neurological damage.
10. Nissl Staining: Morphology and distribution of neurons in the ischemic penumbra following 24 h post-reperfusion were observed using cresyl violet (Nissl) staining (Li et al., 2023). The experimental procedure strictly followed the manufacturer’s instructions for cresyl violet dye. Observations and photographs were made using a Leica microscope.
11. RT-qPCR: Total RNA was extracted from cells and brains using TRIzol reagent. Reverse transcription was performed with RT-qPCR according to the HiScript II Q RT SuperMix for qPCR (+gDNA wiper) kit with the Taq Pro Universal SYBR qPCR Master Mix, reverse transcription with RT-qPCR was performed. RT-qPCR reactions were run on a Bio-Rad CFX96. The 18S gene was considered as an internal reference. The relative expression levels were assessed using the 2^−ΔΔCT^ method. Primer sequences are detailed in Supplementary Table S2.
12. Western blot: The samples underwent separation through either 10% or 12.5% SDS-PAGE gel electrophoresis and were subsequently moved into PVDF membranes. Following this, the membranes were immersed in 5% skim milk for a duration of 2 h and then probed with anti-CD63 mouse monoclonal, anti-TSG101 rabbit monoclonal, rabbit monoclonal anti-KLF14, anti-NF-κB p65 rabbit monoclonal, anti-phosphorylation NF-κB p65 rabbit monoclonal, and anti-β-actin mouse monoclonal at 4 °C overnight. Subsequently, membranes were incubated with anti-mouse IgG-HRP or anti-rabbit IgG-HRP for 2 h at room temperature. Protein signals were detected using a highly sensitive ECL chemiluminescence substrate and analyzed with a Tanon 5,200 Multi system.
13. Statistical Analysis: The data is displayed as mean ± SD, unless specified otherwise. Statistical analyses were employed by GraphPad Prism. Statistical analysis among more groups were determined by One-way ANOVA. *p*-value less than 0.05 was considered statistically significant.

## Results

1. Characterization of H-EXOs

**FIGURE 1:**
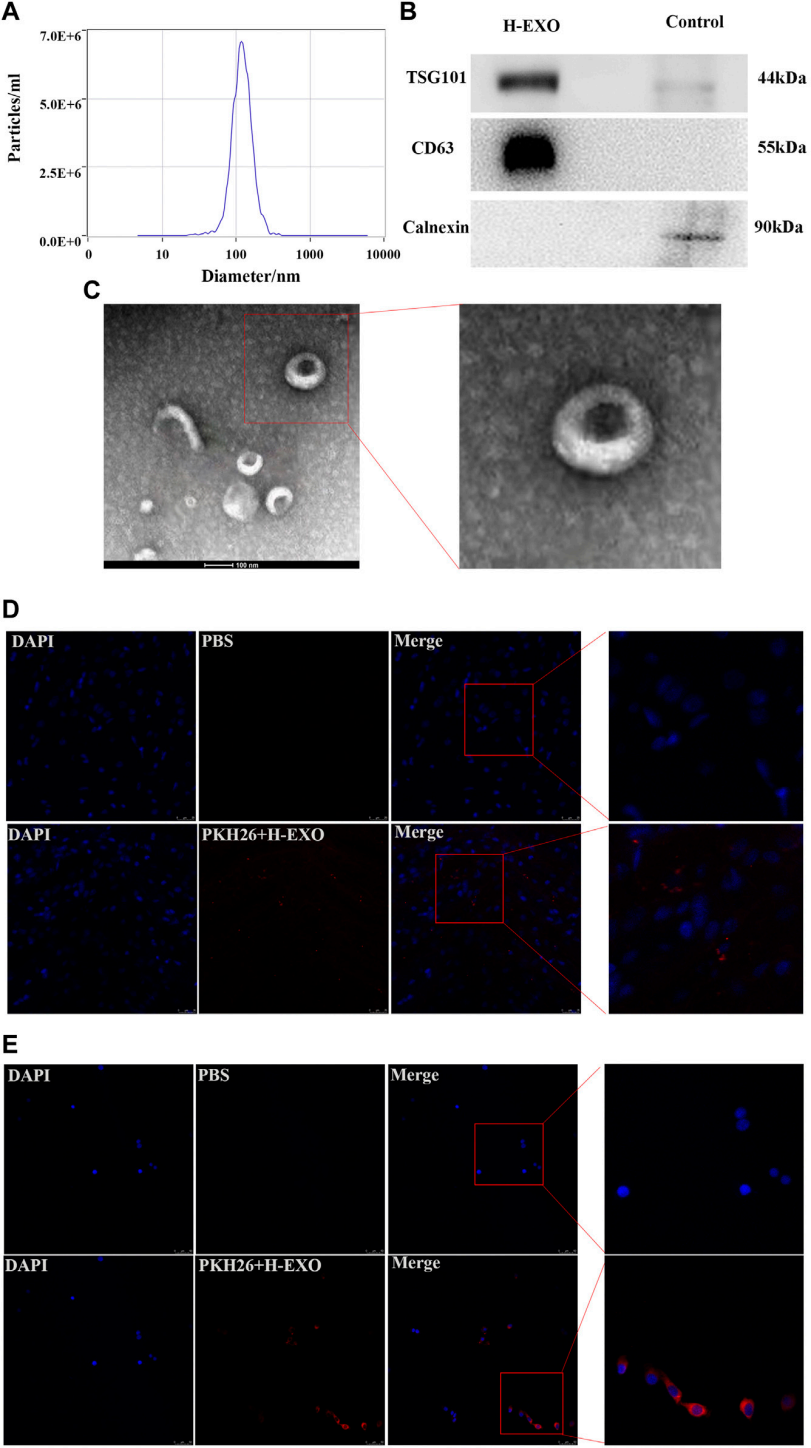
Characterization of H-EXOs and their internalization by cells. H-EXOs can enter MCAO/R-treated brain tissue and can be taken up by HT22 cells. **(A)** NTA of H-EXOs. **(B)** Western blot detection of exosome markers CD63, TSG101 and negative control Calnexin. **(C)** TEM images of exosomes of H-EXOs. Scale bar, 50 nm. **(D)** Immunofluorescence staining showing PKH26-labeled exosomes (red) in brain slice of MCAO-R injured rat. Nuclei stained blue by DAPI. Scale bar, 50 µm. **(E)** Immunofluorescence staining showing PKH26-labeled exosomes (red) in HT22 cells. Cells nucleus were stained with DAPI (blue). Scale bar, 50 µm.

Exosomes are defined as small membrane vesicles with a diameter of 40–140 nm and a circular shape (Simons and Raposo, 2009). H-EXOs in this study are within a median diameter of 118 nm and the size distribution is shown in Figure 1A. The concentration of this isolated exosomes are about 1.1×10^14^ particles/mL. Exosomes should express transmembrane proteins such as CD63 and endosome proteins such as TSG101 (Lötvall et al., 2014), and we confirmed that our isolated exosomes H-EXOs expressed both CD63 and TSG101, meanwhile our results showed that the negative control Calnexin was not expressed in H-EXOs (Figure 1B). Purified H-EXOs were examined using transmission electron microscopy (TEM), which revealed their typical cup-shaped and clear lipid bilayer structure (Figure 1C).

Because the interaction between H-EXOs and their target tissues or cells is important for the therapeutic effect (van Niel et al., 2018), so we tested whether H-EXOs are taken up by ischemia-reperfusion injured brain tissues of rats and HT22 cells. HT22 is a sub-line derived from parent HT4 cells that were originally immortalized from cultures of primary mouse hippocampal neurons, which is widely used as an ideal hippocampal neuronal model (Zaulyanov et al., 1999; Pascual et al., 2012). MCAO/R-injured rats were injected with 1 mL PKH26-labeled H-EXOs from tail vein at the same time of reperfusion. Four hours later, the rats were sacrificed and the brain tissues were sampled. Confocal microscopy analysis revealed no red fluorescence in the brain tissue sections of the control rats. However, red fluorescence was evident in the brain tissue sections of the rats from the experimental group (PKH26-labeled H-EXO). Thus, PKH26-labeled H-EXOs could reach the brain tissue of rats after MCAO/R injury (Figure 1D). Furthermore, the PKH26-labeled H-EXOs were added to the wells of HT22 cells for 12 h. The labeled exosomes were found in HT22 cells and located in the cytoplasm, indicating their complete internalization. As showed in Figure 1E, almost all HT22-cells became positive for PKH26. These results suggested that our isolated H-EXOs are able to enter the penumbra region of the cerebral cortex of rats after tMCAO operation and be taken up by cultured HT22 cells.

Taken together, we successfully isolated H-EXOs for the following study.

2. H-EXOs significantly inhibited the production of pro-inflammatory molecules and protected neurons from MCAO/R injury in rats

**FIGURE 2:**
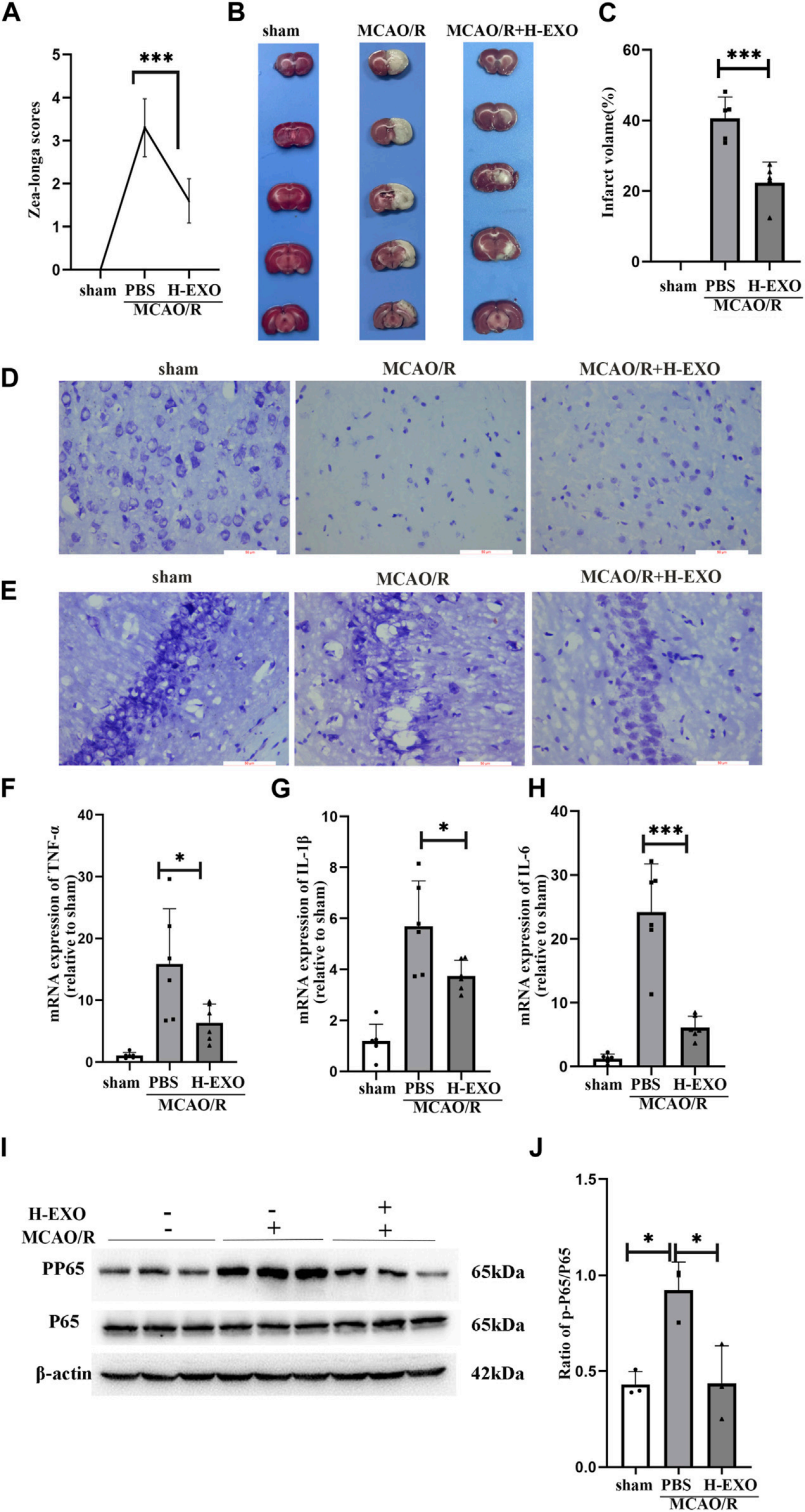
H-EXOs attenuate brain damage and inhibit inflammation in the right cortical ischemic penumbral areas of MCAO2h/R24h rats. **(A)** Neurological deficit scores of rats with MCAO/R injury in different groups. H-EXOs were administrated at the time of reperfusion. Neurological deficits were evaluated using the Longa score 24h after reperfusion (n = 10). **(B)** Coronal brain sections were stained by TTC in the sham group, MCAO/R group, and H-EXOs group, along with quantification of infarct volumes **(C)** (n = 5). **(D)** Microscopic images of Nissl-stained rat brain cortex. Scale bar, 100 μm. **(E)** Microscopic images of Nissl-stained rat brain hippocampal CA1 region. Scale bar, 100 μm. **(F–H)** RT-qPCR detect the level of TNF-α, IL-1β, and IL-6 in the ischemic penumbral area of the right cerebral cortex of MCAO2h/R24h rats after tail vein injection with H-EXOs for 24h (n = 5). **(I)** Western blot detection of NFκB P65 and PP65 levels in the ischemic penumbral area of the right cerebral cortex of MCAO2h/R24h rats after tail vein injection of H-EXOs for 24h. **(J)** Quantification of western blot results in **(J)** (n = 3). The data underwent analysis using a one-way ANOVA method. *p < 0.05, ***p < 0.001.

First, to validate the protective effect of H-EXOs treatment against brain ischemia, we performed MCAO surgery in rats, as previously described. Ten rats were randomly assigned to the sham (received the same procedure as MCAO but without artery occlusion), MCAO/R, or MCAO/R + H-EXOs group. After 24 h of reperfusion, neurological deficits were examined by the Longa’s score. We found that the MCAO/R group had a score of about 3, while the group treated with H-EXOs had neurological scores between 1 and 2 (Figure 2A). Subsequently, TTC staining of the rat brain was performed to determine infarct volume. As shown in Figure 2B, the red area is normal brain tissue, while the white area is infarcted due to ischemia. The infarct area in the MCAO/R group accounted for about 40% of the total brain volume, while that in the H-EXOs group accounted for about 20% of the total brain volume (Figure 2C), indicating that H-EXOs remarkably reduced cerebral infarction volume. Previous studies have suggested that ischemic-vulnerable areas include the hippocampus, cortex, thalamus, and other parts. Among various types of cells, neurons have the highest sensitivity to ischemia. In order to confirm whether H-EXOs could reduce neuronal damage from MCAO/R injury, morphological changes in neurons were investigated by Nissl staining. We found that the MCAO/R group had collapsed neuronal cell structure, the presence of vacuoles around cells, nuclear condensation, and reduced Nissl bodies. In contrast, most of the neurons from the H-EXO-treated group showed a relatively intact morphology, with only a few cells displaying nuclear condensation and reduced Nissl bodies (Figure 2D). Similar changes were also observed in the CA1 region of the rat hippocampus, which is enriched with neurons (Figure 2E). All the above results suggested that H-EXOs can protect neurons from ischemia-reperfusion injury.

Next, to explore the underlying mechanisms of the protective effects of H-EXOs on rat brain tissues during ischemic-reperfusion injury, we assessed the expression of pro-inflammatory cytokines IL-1β, IL-6, and TNF-α in penumbra areas of cerebral cortex of MCAO/R rats by RT-qPCR. The results showed that the mRNA levels of IL-1β, IL-6, and TNF-α in rat brains after MCAO/R injury were apparently higher than those in the brains of the sham-operated rats, and H-EXOs treatment reduced the increased levels of these factors (Figures 2F–H). The NF-κB signaling pathway is an important inflammation pathway (Zhao et al., 2021). When stroke occurs, the NF-κB inflammatory signaling pathway is activated to stimulate the phosphorylation of P65 protein to p-P65, and then p-P65 enters the nucleus to further induce the release of inflammatory factors including TNF-α, IL-1β and IL-6 etc. (Singh and Singh, 2020). Therefore, we examined the phosphorylation of P65 in the penumbra region of the cerebral cortex in rats. The level of P65 phosphorylation was significantly increased after MCAO/R but suppressed by H-EXOs (Figures 2I, J). In summary, H-EXOs can effectively alleviate the production of inflammatory factors and protect neurons in brain tissue of rats from MCAO/R injury.

3. Internalized H-EXOs dramatically reduced the production of pro-inflammatory factors and protected cultured HT22 neuronal cells from OGD/R injury

**FIGURE 3:**
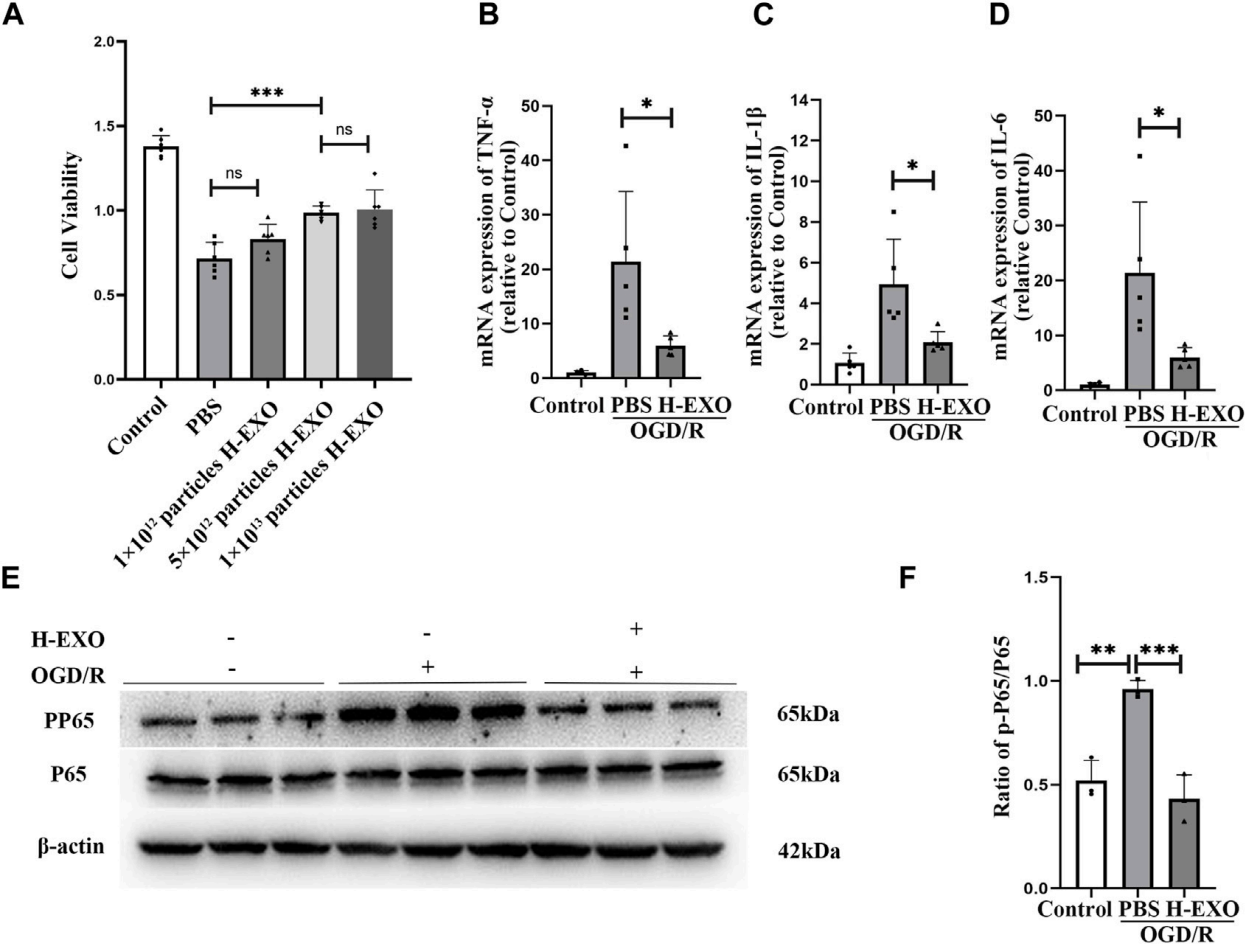
H-EXOs prevent cultured HT22 neuronal cells from OGD/R injury. **(A)** The therapeutic effects of different doses of H-EXOs were tested after OGD4h/R12h by using MTT assay (n = 6). **(B–D)** RT-qPCR detect the change of TNF-α, IL-1β, and IL-6 mRNA expression in HT22 cells after OGD/R injury (n = 5). **(E)** Western blot detection of NFκB P65 and p-P65 level in HT22 cells caused by OGD/R injury and its quantification in **(F)** (n = 3). The data underwent analysis using a one-way ANOVA method. *p < 0.05, **p < 0.01, ***p < 0.001.

To confirm the protective effect of H-EXOs on injured neuronal cells, HT22 cells were cultured with conditional medium which contains 1 mM Na_2_S_2_O_4_ and sugar-free medium without FBS for 2 h and then cultured in DMEM medium with 10% FBS for 12 h to construct the OGD/R model (Gao et al., 2020). To verify the therapeutic effect of H-EXOs on OGD/R, OGD/R-treated HT22 cells were incubated with different dosages of H-EXOs. As shown in Figure 3A, HT22 cell viability was significantly decreased about 50% in the OGD/R group compared with the control group. 1×10^12^ particles/mL of H-EXOs had no obvious therapeutic effect, and 5×10^12^ particles/mL of H-EXOs significantly improved cell viability, but there was no better therapeutic effect when the dosage reached 1×10^13^ particles/mL. Therefore, the optimal effective dosage of H-EXOs was 5×10^12^ particles/mL. RT-qPCR results also confirmed that OGD/R injury significantly increased the production of TNF-α, IL-1β, and IL-6, which were apparently downregulated in the H-EXOs treated-cells (Figures 3B–D). We also measured the P65/p-P65 protein levels in HT22 cells and found that H-EXOs treatment markedly reduced the protein expression of phosphorylated P65 (p-P65) induced by OGD/R (Figures 3E, F). These results indicated that H-EXOs inhibit OGD/R-induced inflammatory responses in HT22 cells.

4. The expression of KLF14 was increased by H-EXOs, which turns out to be a protective factor produced during cerebral ischemia-reperfusion injury

**FIGURE 4:**
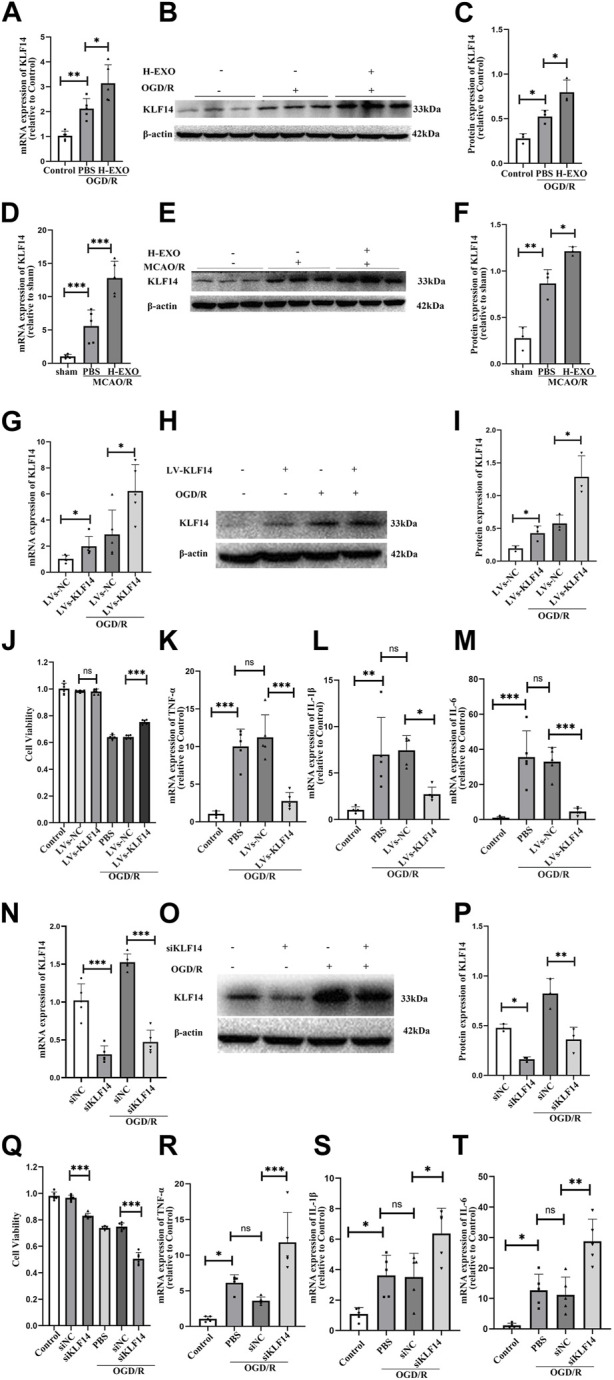
H-EXOs can induce KLF14 expression which is an endogenous regulator involved in the pathophysiology of the inflammatory response in ischemic stroke. **(A)** RT-qPCR analysis KLF14 mRNA change level of HT22 cells caused by OGD/R injury (n = 5). **(B)** Western blot analysis of KLF14 protein expression in HT22 cells after injury. **(C)** Statistical analysis of **(B)** (n = 3). **(D)** RT-qPCR analysis of KLF14 level in the right cortical ischemic penumbral areas of MCAO2/R24h rats after H-EXOs treatment (n = 5). **(E)** Western blot detection of KLF14 level in the right cortical ischemic penumbral areas of MCAO2h/R24h rats after H-EXOs treatment and its quantification in **(F)** (n = 3). **(G)** RT-qPCR detection of KLF14 expression in HT22 cells treated with LVs-KLF14 and LVs-NC after OGD4h/R12h (n = 5). **(H)** Western blot detection of KLF14 expression in HT22 cells after transfection with LVs-KLF14 and LVs-NC after OGD4h/R12h and its quantification in **(I)** (n = 3). **(J)** MTT assay in HT22 cells treated with LVs-KLF14 and LVs-NC after OGD4h/R12h (n = 6). **(K–M)** RT-qPCR detect the TNF-α, IL-6, and IL-1β expression level of HT22 cells treated with LVs-KLF14 and LVs-NC after OGD4h/R12h (n = 5). (N) RT-qPCR test KLF14 expression level in HT22 cells treated with siKLF14 and siNC after OGD4h/R12h (n = 5). **(O)** Western blot detection of KLF14 expression in HT22 cells after siKLF14 and siNC transfected after OGD4h/R12h and its quantification in **(P)** (n = 3). **(Q)** Cell viability of HT22 cells treated with siKLF14 and siNC after OGD4h/R12h were detected using MTT assay. (n = 6). **(R–T)** RT-qPCR detect TNF-α, IL-6 and IL-1β change level in HT22 cells treated with siKLF14 and siNC after OGD4h/R12h (n = 5). The data underwent analysis using a one-way ANOVA method. *p < 0.05, **p < 0.01, ***p < 0.001.

Transcription factor KLF14 is a member of the KLF family and exerts anti-inflammatory effects in many diseases, such as sepsis, atherosclerosis, and immune-mediated hepatic injury, etc. We wonder whether the anti-inflammatory effects of H-EXOs are related to KLF14. First, we tested the expression of KLF14 in the brain tissue of rats and cultured HT22 cells suffering from I/R injury. The results of RT-qPCR and Western blot indicated that the expression of KLF14 in the OGD/R group was significantly increased compared with that in the control group, and the expression of KLF14 in the H-EXOs group was further increased, about a 1.5-fold increase compared with the OGD/R group (Figures 4A–C). Similar results were observed *in vivo* experiments. H-EXOs significantly increased the levels of KLF14 mRNA and protein in the ischemic penumbra region of the cerebral cortex, respectively, about 2.5-fold and 1.5-fold compared with the MCAO/R group (Figures 4D–F). Summarizing the results given above, KLF14 expression was significantly increased in brain tissue and cultured cells after injury, and further increased with H-EXOs treatment.

Next, to confirm whether the increase of KLF14 expression is potentially a protective factor or a pathogenic factor, we regulated the expression of KLF14 in HT22 cells by using the lentivirus vector KLF14 (LVs-KLF14) for overexpressing and a small-interfering RNA (siKLF4) for downregulation. After transfection, we detected the HT22 cell viability and the expression of pro-inflammatory factors. Compared with the LVs-NC group, the expression levels of KLF14 mRNA and protein significantly increased about 2-fold both in the normal cultured and OGD/R-injured HT22 cell groups with LVs-KLF14, indicating that overexpressing KLF14 transfection was successful (Figures 4G–I). Even though overexpressing KLF14 did not affect normal cell viability, the survival of OGD damaged cells was promoted by KLF14 overexpression (Figure 4J). Meanwhile, the results of RT-qPCR showed that there was no significant difference in the expression levels of pro-inflammatory factors *TNF-α*, *IL-1β*, and *IL-6* between the LVs-KLF14 group and the LVs-NC group under non-OGD/R culture conditions (Supplementary Figure S1A). However, after OGD/R treatment, the expression level of pro-inflammatory factors in the LVs-KLF14 group was dramatically lower over 50% than that in the LVs-NC group (Figure 4K–M). These results suggested that overexpressing KLF14 can suppress the expression of pro-inflammatory factors. To further confirm the anti-inflammatory effect of KLF14, we also used a small-interfering RNA (siKLF4) to repress KLF14 expression in HT22 cells, and the cells treated with siKLF14 exhibited lower KLF14 mRNA and protein expression (about 65% reduction) both in the normal cultured and OGD/R-injured HT22 cells groups (Figure 4N–P). The results of the MTT assay demonstrated that the cell viability of siKLF14 transfected cells was lower than that of siNC transfected cells (about 15% reduction). After OGD/R injury, the cell viability of siKLF14 transfected cells was much lower than that of the siNC transfected group (about 30% reduction, Figure 4O). The results of RT-qPCR exhibited no significant difference in the expression levels of pro-inflammatory factors from the siKLF14 group and the siNC group (Supplementary Figure S1B). However, after OGD/R, cells transfected with siKLF14 showed higher levels of pro-inflammatory factors than the cells transfected with siNC (about two to three fold increase, Figure 4P).

In conclusion, KLF14 is a protective factor that is produced during cerebral ischemia-reperfusion injury. H-EXOs treatment increased KLF14 expression after I/R injury.

5. H-EXOs played anti-inflammatory roles by upregulating KLF14

**FIGURE 5:**
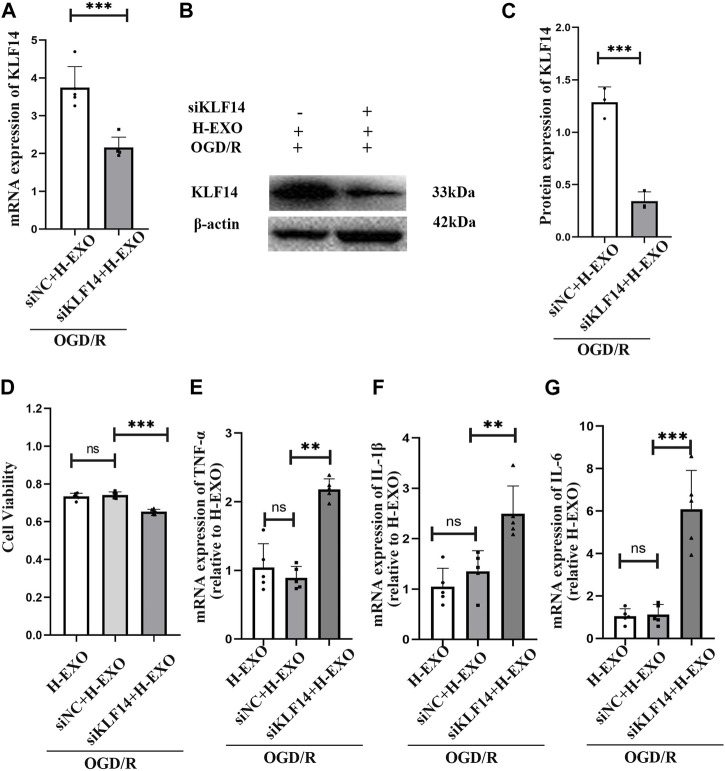
Interference with KLF14 alleviates H-EXOs-mediated anti-inflammatory effects. **(A)** RT-qPCR detection of KLF14 levels in HT22 neuronal cells after treatment with OGD/R + H-EXOs + siNC or OGD/R + H-EXOs + siKLF4 (n = 5). -- **(B)** Western blot detection of KLF14 levels in HT22 neuronal cells after treatment with OGD/R + H-EXOs + siNC or OGD/R + H-EXOs + siKLF4 and its quantification in **(C)** (n = 3). **(D)** MTT assay assessing cell viability in HT22 cells after treatment with siKLF4 and siNC following OGD4h/R12h and H-EXOs treatment (n = 5). **(E–G)** RT-qPCR detection of TNF-α, IL-1β, and IL-6 mRNA expression in HT22 cells after treatment with siKLF4 and siNC following OGD4h/R12h and H-EXOs treatment (n = 5). All the data were analyzed by one‐way ANOVA. *p < 0.05, **p < 0.01, ***p < 0.001.

To investigate whether KLF14 was related to the anti-inflammatory effects of H-EXOs, we regulated KLF14 expression with siKLF4 transfection in OGD/R-injured HT22 neural cells and then incubated them with H-EXOs. We found that, with siKLF14 treatment, the expression of KLF14 mRNA and protein induced by H-EXOs separately decreased to 60% and 40%, compared with the siNC treatment (Figures 5A–C). To better confirm whether KLF14 is involved in mediating the anti-inflammation effect of H-EXOs, we tested cell viability by the MTT assay and pro-inflammatory factors expression by RT-qPCR. The results showed that siKLF4 transfected cells with H-EXOs treatment presented cell viability decreased to 85% and pro-inflammatory cytokines expression increased to 2.5-fold of H-EXO-treated siNC cells (Figures 5D–G). Collectively, H-EXOs exerted anti-inflammatory effects in neurons after ischemic stroke via upregulating the expression of KLF14.

## Discussion

Inflammatory response is a hallmark of ischemic stroke onset, especially after reperfusion, which initiates rapidly and lasts for a long time, thereby providing a long window period for treatment (Justin et al., 2018; Kelly et al., 2018). Therefore, understanding the mechanism of post-stroke inflammation and regulating it will be a highly potential stroke treatment strategy (Shi et al., 2021). An increasing number of studies nowadays demonstrated the vital role of exosomes in maintaining the ischemic injured brain structure and function, given their unique lipid bilayer structure and diverse cargos (He et al., 2018). Here, we showed that H-EXOs protected the brain from tMCAO/R injury in rats and OGD/R injury in cultured neuronal cells through the suppression of the inflammatory response, suggesting that H-EXOs is a promising therapeutic biological agent for I/R injury. Although several recent studies have indicated the potential therapeutic effects of exosomes derived from post-ischemic HUVECs in the treatment of ischemic stroke, more studies are warranted to explore new directions. Some researchers believe that the different times of ischemic preconditioning may result in varying metabolomics differences (Baranovicova et al., 2020). The role of H-EXOs without pretreatment remains unconfirmed, which contains different factors and chemical substances. Thus, herein, we explored the role and underlying mechanism of H-EXOs on cerebral inflammation in I/R injured rats.

The increased levels of IL-1β, TNF-α and IL-6 and the phosphorylation of P65 are the key phenomena that reflect the degree of injury caused by an inflammatory response (Li et al., 2014; Wang et al., 2020). Ischemic stroke results in an increase in the secretion of inflammatory factors, which activates the NF-κB pathway and promotes P65 phosphorylation, thereby further stimulating the production of inflammatory factors (Kong et al., 2022; Luo et al., 2022). In our study, the levels of IL-1β, TNF-α and IL-6, and the phosphorylation of P65 were significantly increased in the rat brain tissue and cultured HT22 cells after ischemia-reperfusion injury (Figures 2F–H; Figures 3B–D), which is consistent with other researchers’ findings (Althurwi et al., 2022). H-EXOs significantly inhibited the upregulation of these inflammatory factors’ expression. Previous studies have demonstrated that neurons, especially in the CA1 area of the hippocampus, are particularly sensitive to ischemic damage. Nissl staining was performed to assess the morphological changes present in the injured areas of the rat brain. Our result indicated that most neurons from the H-EXO-treated rats showed a relatively intact morphology, with only a few cells displaying nuclear condensation and reduced Nissl bodies, compared with those from the rats without H-EXO-treatment (Figure 2D). Collectively, we confirmed that H-EXOs could protect neurons from ischemia-reperfusion injury through the suppression of the inflammatory response.

We wondered the underlying mechanism of the anti-inflammatory action of H-EXOs involved in I/R injury. Extensive evidence suggests that the role of exosomes in ischemic stroke may be associated with the function as messengers in intercellular communication. During the treatment process for cerebral ischemia, exosomes secrete a variety of factors and modulate the expression of endogenous target genes (Cheng et al., 2021; Guo et al., 2021; Huang et al., 2021). Therefore, we wanted to know the factors that are involved in the anti-inflammatory effect of H-EXOs. We have previously shown that transcription factor KLF14 expression is upregulated in primary neurons after OGD45min/R6h injury (Shi et al., 2017). KLF14, as one of the KLF family members, which is widely expressed in many tissues and involved in the regulation of many cellular processes, including embryogenesis, proliferation, differentiation, apoptosis, etc. (Chen et al., 2020). KLF14 has a role in immune regulation, but has different regulatory functions in different disease states (Xie et al., 2017; Du et al., 2021), and the reduction of KLF14 expression was found to increase the expression of inflammatory factors TNF-α, IL-1β, and IL-6. This effect could be reversed by KLF14 overexpression (Hu et al., 2018; Chen et al., 2019; Yuan et al., 2022). Based on the above, we wonder whether the anti-inflammatory effects of H-EXOs are related to KLF14. In the present study, we observed an increase in the expression of KLF14 in the rat brain tissue and HT22 cells after I/R injury (Figures 4A–F). The role of KLF14 in cerebral ischemic injury has not yet been studied. To fill the blank, we overexpressed and downregulated the expression levels of KLF14 in OGD/R treated HT22 cells.The results indicated that KLF14 overexpression improved cell viability and suppressed the inflammatory response of HT22 cells after OGD/R (Figure 4J–M). Meanwhile KLF14 downregulation by using siRNA led to a reduction in cell viability and an elevated inflammatory response (Figure 4Q–T). Taken together, KLF14 is involved in the pathological process of brain I/R injury and exerts protective effects by inhibiting the production of pro-inflammatory factors. This finding underscores the pathological mechanism of ischemic stroke and provides a new therapeutic strategy.

In this study, the treatment with H-EXOs after I/R injury further increased the expression of KLF14 (Figures 4A–F). Neurons are susceptible to damage after ischemic stroke, and it is of great importance to study them, so in the present study we examined the changes in KLF14 expression in neuronal cells after ischemia-reperfusion injury. Whether there are changes in KLF14 expression in other types of cells needs to be explored in more experiments. The increased KLF14 in H-EXOs treated cells may come from the exosomes or from their induction. To give an explicit answer, we tested the protein level of KLF14 in H-EXOs but could not observe any strong signal (Supplementary Figure S2), which may suggest that H-EXOs induced the expression of KLF14 in neuronal cells after I/R injury, and whether there is more KLF14 protein expression in the exosomes secreted by ischemia preconditioned HUVEC needs to be further investigated. Besides, siKLF14 reversed the anti-inflammatory effect of H-EXOs (Figures 5D–G). Overall, H-EXOs exerted anti-inflammatory effects in neurons after ischemic stroke via upregulating the expression of KLF14.

In the present study, we investigated that H-EXOs could partially inhibit the inflammatory responses after I/R injury. The pathophysiology of brain ischemia-reperfusion injury is terribly complicated, involving various mechanisms, including excitotoxicity, oxidative stress, neuroinflammation, and apoptosis (Qin et al., 2022). It is known that KLF14 overexpression suppresses inflammatory effects by inhibiting P65 transcription (Liu et al., 2022). However, H-EXOs treatment did not change the expression of P65 but inhibited the phosphorylation of P65 in our study. Maybe H-EXOs regulate P65 expression with other mechanisms. Recent studies suggested that the expression of KLF14 can be influenced by the activated transforming growth factor β (TGF-β) pathway (Gonzalez et al., 2013; Chen L. et al., 2023) or microRNAs, and it also can regulate other miRNAs (Li et al., 2020; Chen X. Z. et al., 2023). It is very likely that the miRNAs from H-EXOs regulate KLF14 expression, and this hypothesis will be confirmed by our further study. We will also elucidate whether H-EXOs exert other important effects at different stages of the pathological processes of ischemic stroke. Although exosomes have been proposed as a therapeutic approach and potential biomarkers for stroke (Otero-Ortega et al., 2019; Jafarzadeh-Esfehani et al., 2020; Yang et al., 2021), the clinical application is still limited. Thus, it is important to validate the effects of H-EXOs treatment in other animal models of brain ischemic stroke to promote its clinical application.

In conclusion, our research suggests that KLF14 might be a potential target for the treatment of cerebral ischemic injury, and H-EXOs are promising to be a therapeutic strategy for ischemic stroke.

## Data Availability

The original contributions presented in the study are included in the article/Supplementary Materials, further inquiries can be directed to the corresponding authors.

## References

[B1] Althurwi H. N. Abdel-Rahman R. F. Soliman G. A. Ogaly H. A. Alkholifi F. K. Abd-Elsalam R. M. (2022). Protective effect of beta-carotene against myeloperoxidase- mediated oxidative stress and inflammation in rat ischemic brain injury. Antioxidants (Basel) 11 (12), 2344. 10.3390/antiox11122344 36552554 PMC9774247

[B2] Baranovicova E. Kalenska D. Tomascova A. Holubcikova S. Lehotsky J. (2020). Time-related metabolomics study in the rat plasma after global cerebral ischemia and reperfusion: effect of ischemic preconditioning. IUBMB Life 72 (9), 2010–2023. 10.1002/iub.2340 32663378

[B3] Chen L. Sha M. L. Chen F. T. Jiang C. Y. Li D. Xu C. L. (2023a). Upregulation of KLF14 expression attenuates kidney fibrosis by inducing PPARα-mediated fatty acid oxidation. Free Radic. Biol. Med. 195, 132–144. 10.1016/j.freeradbiomed.2022.12.096 36584797

[B4] Chen X. Shi W. Zhang H. (2020). The role of KLF14 in multiple disease processes. Biofactors 46 (2), 276–282. 10.1002/biof.1612 31925990

[B5] Chen X. Tan Q. Wang Y. Lv H. Wang Z. Lin Z. (2019). Overexpression of KLF14 protects against immune-mediated hepatic injury in mice. Lab. Invest. 99 (1), 37–47. 10.1038/s41374-018-0134-4 30254317

[B6] Chen X. Z. He W. X. Luo R. G. Xia G. J. Zhong J. X. Chen Q. J. (2023b). KLF14/miR-1283/TFAP2C axis inhibits HER2-positive breast cancer progression via declining tumor cell proliferation. Mol. Carcinog. 62 (4), 532–545. 10.1002/mc.23505 36752341

[B7] Cheng C. Chen X. Wang Y. Cheng W. Zuo X. Tang W. (2021). MSCs-derived exosomes attenuate ischemia-reperfusion brain injury and inhibit microglia apoptosis might via exosomal miR-26a-5p mediated suppression of CDK6. Mol. Med. 27 (1), 67. 10.1186/s10020-021-00324-0 34215174 PMC8254277

[B8] Ding W. Gu Q. Liu M. Zou J. Sun J. Zhu J. (2023). Astrocytes-derived exosomes pre-treated by berberine inhibit neuroinflammation after stroke via miR-182-5p/Rac1 pathway. Int. Immunopharmacol. 118, 110047. 10.1016/j.intimp.2023.110047 36996739

[B9] Du Z. Liu M. Wang Z. Lin Z. Feng Y. Tian D. (2021). EZH2-mediated inhibition of KLF14 expression promotes HSCs activation and liver fibrosis by downregulating PPARγ. Cell Prolif. 54 (7), e13072. 10.1111/cpr.13072 34031939 PMC8249795

[B10] Ferguson S. Kim S. Lee C. Deci M. Nguyen J. (2018). The phenotypic effects of exosomes secreted from distinct cellular sources: a comparative study based on miRNA composition. Aaps J. 20 (4), 67. 10.1208/s12248-018-0227-4 29713834 PMC6461218

[B11] Gao Y. Cao X. Zhang X. Wang Y. Huang H. Meng Y. (2020). Brozopine inhibits 15-LOX-2 metabolism pathway after transient focal cerebral ischemia in rats and OGD/R-Induced hypoxia injury in PC12 cells. Front. Pharmacol. 11, 99. 10.3389/fphar.2020.00099 32153408 PMC7047151

[B12] Gonzalez C. R. Vallcaneras S. S. Calandra R. S. Gonzalez Calvar S. I. (2013). Involvement of KLF14 and egr-1 in the TGF-beta1 action on Leydig cell proliferation. Cytokine 61 (2), 670–675. 10.1016/j.cyto.2012.12.009 23317878

[B13] Guo L. Huang Z. Huang L. Liang J. Wang P. Zhao L. (2021). Surface-modified engineered exosomes attenuated cerebral ischemia/reperfusion injury by targeting the delivery of quercetin towards impaired neurons. J. Nanobiotechnology 19 (1), 141. 10.1186/s12951-021-00879-4 34001136 PMC8130330

[B14] He C. Zheng S. Luo Y. Wang B. (2018). Exosome theranostics: biology and translational medicine. Theranostics 8 (1), 237–255. 10.7150/thno.21945 29290805 PMC5743472

[B15] Hu W. Lu H. Zhang J. Fan Y. Chang Z. Liang W. (2018). Krüppel-like factor 14, a coronary artery disease associated transcription factor, inhibits endothelial inflammation via NF-κB signaling pathway. Atherosclerosis 278, 39–48. 10.1016/j.atherosclerosis.2018.09.018 30248551 PMC6441279

[B16] Huang Z. Guo L. Huang L. Shi Y. Liang J. Zhao L. (2021). Baicalin-loaded macrophage-derived exosomes ameliorate ischemic brain injury via the antioxidative pathway. Mater Sci. Eng. C Mater Biol. Appl. 126, 112123. 10.1016/j.msec.2021.112123 34082940

[B17] Jafarzadeh-Esfehani R. Soudyab M. Parizadeh S. M. Jaripoor M. E. Nejad P. S. Shariati M. (2020). Circulating exosomes and their role in stroke. Curr. Drug Targets 21 (1), 89–95. 10.2174/1389450120666190821153557 31433753

[B18] Justin A. Divakar S. Ramanathan M. (2018). Cerebral ischemia induced inflammatory response and altered glutaminergic function mediated through brain AT(1) and not AT(2) receptor. Biomed. Pharmacother. 102, 947–958. 10.1016/j.biopha.2018.03.164 29710550

[B19] Kalluri R. LeBleu V. S. (2020). The biology, function, and biomedical applications of exosomes. Science 367 (6478), eaau6977. 10.1126/science.aau6977 32029601 PMC7717626

[B20] Kelly P. J. Murphy S. Coveney S. Purroy F. Lemmens R. Tsivgoulis G. (2018). Anti-inflammatory approaches to ischaemic stroke prevention. J. Neurology, Neurosurg. Psychiatry 89 (2), 211–218. 10.1136/jnnp-2016-314817 28935831

[B21] Kong L. Li W. Chang E. Wang W. Shen N. Xu X. (2022). mtDNA-STING Axis mediates microglial polarization via IRF3/NF-κB signaling after ischemic stroke. Front. Immunol. 13, 860977. 10.3389/fimmu.2022.860977 35450066 PMC9017276

[B22] Kunz A. Dirnagl U. Mergenthaler P. (2010). Acute pathophysiological processes after ischaemic and traumatic brain injury. Best Pract. Res. Clin. Anaesthesiol. 24 (4), 495–509. 10.1016/j.bpa.2010.10.001 21619862

[B23] Li D. He T. Zhang Y. Liu J. Zhao H. Wang D. (2023). Melatonin regulates microglial polarization and protects against ischemic stroke-induced brain injury in mice. Exp. Neurol. 367, 114464. 10.1016/j.expneurol.2023.114464 37301531

[B24] Li N. Eljaafari A. Bensoussan D. Wang Y. Latger-Cannard V. Serrurier B. (2006). Human umbilical vein endothelial cells increase *ex vivo* expansion of human CD34(+) PBPC through IL-6 secretion. Cytotherapy 8 (4), 335–342. 10.1080/14653240600845062 16923609

[B25] Li Y. W. Zhang Y. Zhang L. Li X. Yu J. B. Zhang H. T. (2014). Protective effect of tea polyphenols on renal ischemia/reperfusion injury via suppressing the activation of TLR4/NF-κB p65 signal pathway. Gene 542 (1), 46–51. 10.1016/j.gene.2014.03.021 24630969

[B26] Li Z. Yao H. Wang S. Li G. Gu X. (2020). CircTADA2A suppresses the progression of colorectal cancer via miR-374a-3p/KLF14 axis. J. Exp. Clin. Cancer Res. 39 (1), 160. 10.1186/s13046-020-01642-7 32799891 PMC7429896

[B27] Liu C. Yang T. H. Li H. D. Li G. Z. Liang J. Wang P. (2023). Exosomes from bone marrow mesenchymal stem cells are a potential treatment for ischemic stroke. Neural Regen. Res. 18 (10), 2246–2251. 10.4103/1673-5374.369114 37056144 PMC10328279

[B28] Liu L. Yuan Y. Zhou Y. Yao L. Li J. Chen F. (2022). The transcription factor KLF14 attenuates LPS-induced acute lung injury by ameliorating apoptosis of alveolar epithelial cells in mice. Mol. Immunol. 152, 67–77. 10.1016/j.molimm.2022.10.002 36279660

[B29] Liu X. Zhang M. Liu H. Zhu R. He H. Zhou Y. (2021). Bone marrow mesenchymal stem cell-derived exosomes attenuate cerebral ischemia-reperfusion injury-induced neuroinflammation and pyroptosis by modulating microglia M1/M2 phenotypes. Exp. Neurol. 341, 113700. 10.1016/j.expneurol.2021.113700 33741350

[B30] Longa E. Z. Weinstein P. R. Carlson S. Cummins R. (1989). Reversible middle cerebral artery occlusion without craniectomy in rats. Stroke 20 (1), 84–91. 10.1161/01.str.20.1.84 2643202

[B31] Lötvall J. Hill A. F. Hochberg F. Buzás E. I. Di Vizio D. Gardiner C. (2014). Minimal experimental requirements for definition of extracellular vesicles and their functions: a position statement from the International Society for Extracellular Vesicles. J. Extracell. Vesicles 3, 26913. 10.3402/jev.v3.26913 25536934 PMC4275645

[B32] Luo L. Liu M. Fan Y. Zhang J. Liu L. Li Y. (2022). Intermittent theta-burst stimulation improves motor function by inhibiting neuronal pyroptosis and regulating microglial polarization via TLR4/NFκB/NLRP3 signaling pathway in cerebral ischemic mice. J. Neuroinflammation 19 (1), 141. 10.1186/s12974-022-02501-2 35690810 PMC9188077

[B33] Mahemuti Y. Kadeer K. Su R. Abula A. Aili Y. Maimaiti A. (2023). TSPO exacerbates acute cerebral ischemia/reperfusion injury by inducing autophagy dysfunction. Exp. Neurol. 369, 114542. 10.1016/j.expneurol.2023.114542 37717810

[B34] Otero-Ortega L. Laso-García F. Gómez-de Frutos M. Fuentes B. Diekhorst L. Díez-Tejedor E. (2019). Role of exosomes as a treatment and potential biomarker for stroke. Transl. Stroke Res. 10 (3), 241–249. 10.1007/s12975-018-0654-7 30105420

[B35] Pan Q. Wang Y. Liu J. Jin X. Xiang Z. (2023). MiR-17-5p mediates the effects of ACE2-enriched endothelial progenitor cell-derived exosomes on ameliorating cerebral ischemic injury in aged mice. Mol. Neurobiol. 60 (6), 3534–3552. 10.1007/s12035-023-03280-4 36892728

[B36] Parvez S. Kaushik M. Ali M. Alam M. M. Ali J. Tabassum H. (2022). Dodging blood brain barrier with “nano” warriors: novel strategy against ischemic stroke. Theranostics 12 (2), 689–719. 10.7150/thno.64806 34976208 PMC8692911

[B37] Pascual M. Do Couto B. R. Alfonso-Loeches S. Aguilar M. A. Rodriguez-Arias M. Guerri C. (2012). Changes in histone acetylation in the prefrontal cortex of ethanol-exposed adolescent rats are associated with ethanol-induced place conditioning. Neuropharmacology 62 (7), 2309–2319. 10.1016/j.neuropharm.2012.01.011 22349397

[B38] Patel N. Chin D. D. Chung E. J. (2021). Exosomes in atherosclerosis, a double-edged sword: their role in disease pathogenesis and their potential as novel therapeutics. Aaps J. 23 (5), 95. 10.1208/s12248-021-00621-w 34312734

[B39] Peng J. Yu Z. Xiao R. Hu X. Xia Y. (2023). Exosomal ZEB1 derived from neural stem cells reduces inflammation injury in OGD/R-Treated microglia via the GPR30-TLR4-NF-κb Axis. Neurochem. Res. 48 (6), 1811–1821. 10.1007/s11064-023-03866-3 36717511

[B40] Prabhakaran S. Ruff I. Bernstein R. A. (2015). Acute stroke intervention: a systematic review. Jama 313 (14), 1451–1462. 10.1001/jama.2015.3058 25871671

[B41] Qin C. Yang S. Chu Y. H. Zhang H. Pang X. W. Chen L. (2022). Correction To: signaling pathways involved in ischemic stroke: molecular mechanisms and therapeutic interventions. Signal Transduct. Target Ther. 7 (1), 278. 10.1038/s41392-022-01129-1 35961963 PMC9374784

[B42] Shi J. Chen X. Li H. Wu Y. Wang S. Shi W. (2017). Neuron-autonomous transcriptome changes upon ischemia/reperfusion injury. Sci. Rep. 7 (1), 5800. 10.1038/s41598-017-05342-9 28724924 PMC5517505

[B43] Shi K. Zou M. Jia D. M. Shi S. Yang X. Liu Q. (2021). tPA mobilizes immune cells that exacerbate hemorrhagic transformation in stroke. Circulation Res. 128 (1), 62–75. 10.1161/CIRCRESAHA.120.317596 33070717

[B44] Simons M. Raposo G. (2009). Exosomes--vesicular carriers for intercellular communication. Curr. Opin. Cell Biol. 21 (4), 575–581. 10.1016/j.ceb.2009.03.007 19442504

[B45] Singh S. Singh T. G. (2020). Role of nuclear factor kappa B (NF-κB) signalling in neurodegenerative diseases: an mechanistic approach. Curr. Neuropharmacol. 18 (10), 918–935. 10.2174/1570159X18666200207120949 32031074 PMC7709146

[B46] Tang B. Li Y. Xu X. Du G. Wang H. (2024). Electroacupuncture ameliorates neuronal injury by NLRP3/ASC/Caspase-1 mediated pyroptosis in cerebral ischemia-reperfusion. Mol. Neurobiol. 61 (4), 2357–2366. 10.1007/s12035-023-03712-1 37874480

[B47] van Niel G. D’Angelo G. Raposo G. (2018). Shedding light on the cell biology of extracellular vesicles. Nat. Rev. Mol. Cell Biol. 19 (4), 213–228. 10.1038/nrm.2017.125 29339798

[B48] Verweij F. J. Revenu C. Arras G. Dingli F. Loew D. Pegtel D. M. (2019). Live tracking of inter-organ communication by endogenous exosomes *in vivo* . Dev. Cell 48 (4), 573–589. 10.1016/j.devcel.2019.01.004 30745143

[B49] Wan T. Zhong J. Pan Q. Zhou T. Ping Y. Liu X. (2022). Exosome-mediated delivery of Cas9 ribonucleoprotein complexes for tissue-specific gene therapy of liver diseases. Sci. Adv. 8 (37), eabp9435. 10.1126/sciadv.abp9435 36103526 PMC9473578

[B50] Wang K. Ru J. Zhang H. Chen J. Lin X. Lin Z. (2020). Melatonin enhances the therapeutic effect of plasma exosomes against cerebral ischemia-induced pyroptosis through the TLR4/NF-κB pathway. Front. Neurosci. 14, 848. 10.3389/fnins.2020.00848 33013286 PMC7461850

[B51] Wang X. J. Wang M. H. Fu X. T. Hou Y. J. Chen W. Tian D. C. (2018). Selenocysteine antagonizes oxygen glucose deprivation-induced damage to hippocampal neurons. Neural Regen. Res. 13 (8), 1433–1439. 10.4103/1673-5374.235300 30106056 PMC6108205

[B52] Wong C. H. Crack P. J. (2008). Modulation of neuro-inflammation and vascular response by oxidative stress following cerebral ischemia-reperfusion injury. Curr. Med. Chem. 15 (1), 1–14. 10.2174/092986708783330665 18220759

[B53] Xie H. Yao J. Wang Y. Ni B. (2022). Exosome-transmitted circVMP1 facilitates the progression and cisplatin resistance of non-small cell lung cancer by targeting miR-524-5p-METTL3/SOX2 axis. Drug Deliv. 29 (1), 1257–1271. 10.1080/10717544.2022.2057617 35467477 PMC9045767

[B54] Xie W. Li L. Zheng X. L. Yin W. D. Tang C. K. (2017). The role of Krüppel-like factor 14 in the pathogenesis of atherosclerosis. Atherosclerosis 263, 352–360. 10.1016/j.atherosclerosis.2017.06.011 28641818

[B55] Yamaguchi H. Ishii E. Saito S. Tashiro K. Fujita I. Yoshidomi S. (1996). Umbilical vein endothelial cells are an important source of c-kit and stem cell factor which regulate the proliferation of haemopoietic progenitor cells. Br. J. Haematol. 94 (4), 606–611. 10.1046/j.1365-2141.1996.d01-1855.x 8826881

[B56] Yamaguchi H. Ishii E. Tashiro K. Miyazaki S. (1998). Role of umbilical vein endothelial cells in hematopoiesis. Leuk. Lymphoma 31 (1-2), 61–69. 10.3109/10428199809057585 9720715

[B57] Yang L. Qian J. Yang B. He Q. Wang J. Weng Q. (2021). Challenges and improvements of novel therapies for ischemic stroke. Front. Pharmacol. 12, 721156. 10.3389/fphar.2021.721156 34658860 PMC8514732

[B58] Yildirim S. Boehmler A. M. Kanz L. Möhle R. (2005). Expansion of cord blood CD34+ hematopoietic progenitor cells in coculture with autologous umbilical vein endothelial cells (HUVEC) is superior to cytokine-supplemented liquid culture. Bone Marrow Transpl. 36 (1), 71–79. 10.1038/sj.bmt.1705001 15895114

[B59] Yuan Y. Fan G. Liu Y. Liu L. Zhang T. Liu P. (2022). The transcription factor KLF14 regulates macrophage glycolysis and immune function by inhibiting HK2 in sepsis. Cell Mol. Immunol. 19 (4), 504–515. 10.1038/s41423-021-00806-5 34983946 PMC8976055

[B60] Zaulyanov L. L. Green P. S. Simpkins J. W. (1999). Glutamate receptor requirement for neuronal death from anoxia-reoxygenation: an *in vitro* model for assessment of the neuroprotective effects of estrogens. Cell Mol. Neurobiol. 19 (6), 705–718. 10.1023/a:1006948921855 10456232 PMC11545404

[B61] Zhao H. Wu L. Yan G. Chen Y. Zhou M. Wu Y. (2021). Inflammation and tumor progression: signaling pathways and targeted intervention. Signal Transduct. Target Ther. 6 (1), 263. 10.1038/s41392-021-00658-5 34248142 PMC8273155

