# Supplementary material for: Exosomes derived from HUVECs alleviate ischemia-reperfusion induced inflammation in neural cells by upregulating KLF14 expression
Source: Front Pharmacol. 2024 May 2;15:1365928. doi: 10.3389/fphar.2024.1365928 (PMC11096520; doi:10.3389/fphar.2024.1365928)
Supplement: Supplementary file 1 [file Table1.DOCX]

**Table S1 Reagents and materials used in the experiment**

| **Name of reagents and materials** | **Brands** | **Reagent Code** |
| --- | --- | --- |
| HT22 cells | ChaoRui/XiHui Biotech | - |
| HUVEC | Servicebio | STCC12103G |
| DMEM | Gibco | 11965-118 |
| FBS | Gibco | 10099-141 |
| Penicillin - Streptomycin | Gibco | 15140163 |
| PKH26 Red Fluorescent Probe in test Kit | Umibio | UR52302 |
| Na2S2O4 | MacLin | 7775-14-6 |
| sugar-free DMEM | Gibco | 11966025 |
| MTT powder | MacLin | 99839-16-4 |
| SDS powder | Gibco | 28365 |
| Lipofectamine 2000 reagent | Thermo fisher scientific | 12566014 |
| KLF14-siRNA | Ribobio | - |
| KLF14 overexpression lentivirus | Genechem | - |
| Premade poly-L-lysine-coated suture | Cionotech | A4-263650 |
| isoflurane | RWD | R510-22-10 |
| TTC powder | Sigma | T8877 |
| cresyl violet dye | Shyuanye | R20629 |
| TRIzol reagent | Novagen | R401-01 |
| HiScript II Q RT SuperMix for qPCR  (+gDNA wiper) kit | Novagen | R223-01 |
| Taq Pro Universal SYBR qPCR Master Mix | Novagen | Q712-02 |
| RIPA lysis buffer | Beyotime | P0013B |
| BCA | Beyotime | P0012 |
| PVDF membranes | Millipore Sigma | ISEQ00010 |
| skim milk | Beyotime | P0216-300g |
| anti-CD63 mouse monoclonal | Abcam | ab134045 |
| anti-TSG101 rabbit monoclonal | Proteintech | 28283-1-AP |
| anti-Calnexin mouse monoclonal | Santa | sc-23954 |
| rabbit monoclonal anti-KLF14 | ABclonal | A18607 |
| anti-NF-κB p65 rabbit monoclonal | Proteintech | 80979-1-RR |
| anti- phosphorylation NF-κB p65 rabbit monoclonal | Proteintech | 82335-1-RR |
| anti-β-actin mouse monoclonal | Proteintech | 660009-1-lg |
| anti-mouse IgG-HRP | Proteintech | SA00001-1 |
| anti-rabbit IgG-HRP | Proteintech | SA00001-2 |
| highly sensitive ECL chemiluminescence substrate | Biosharp | BL520A |
