# Supplementary figures and images for: Exosomes derived from HUVECs alleviate ischemia-reperfusion induced inflammation in neural cells by upregulating KLF14 expression
Source: Front Pharmacol. 2024 May 2;15:1365928. doi: 10.3389/fphar.2024.1365928 (PMC11096520; doi:10.3389/fphar.2024.1365928)

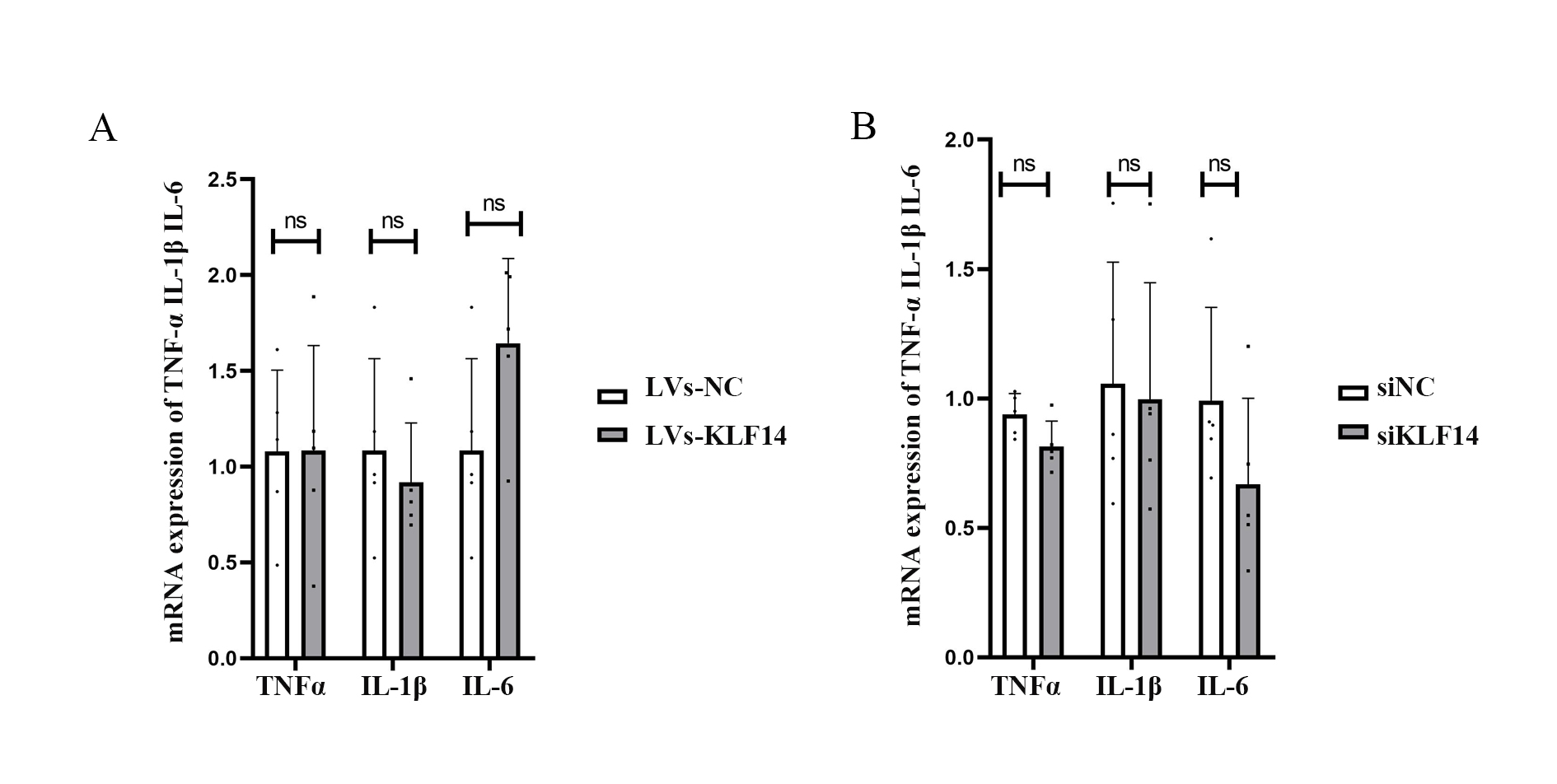

Supplement: Supplementary file 2 [file Image1.JPEG]

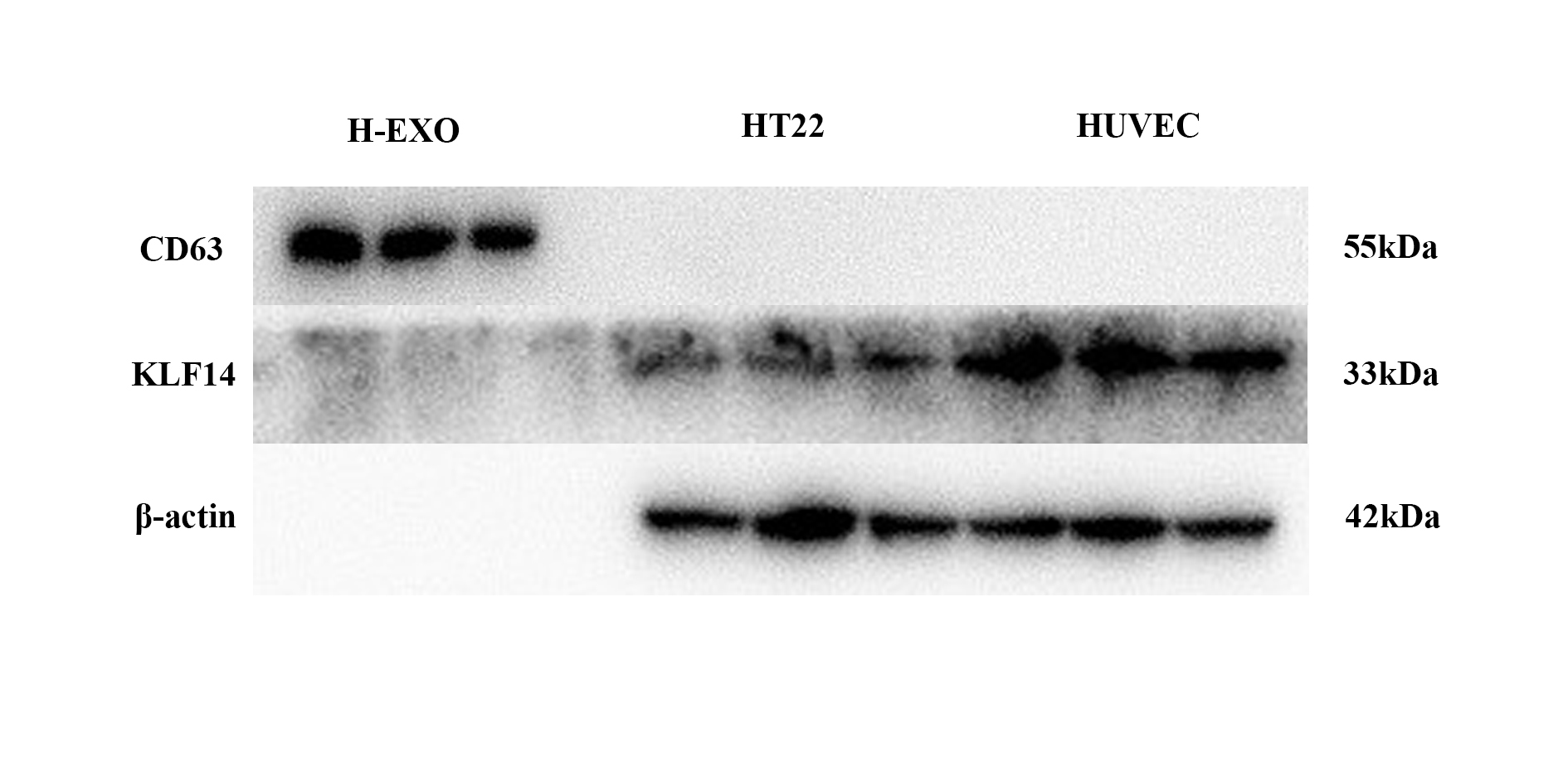

Supplement: Supplementary file 3 [file Image2.JPEG]
