# Supplementary material for: Exosomes derived from HUVECs alleviate ischemia-reperfusion induced inflammation in neural cells by upregulating KLF14 expression
Source: Front Pharmacol. 2024 May 2;15:1365928. doi: 10.3389/fphar.2024.1365928 (PMC11096520; doi:10.3389/fphar.2024.1365928)
Supplement: Supplementary file 4 [file Table2.DOCX]

**Table S2 Primer Sequences**

| **Gene name** | **Forward** | **Reverse** |
| --- | --- | --- |
| 18S | CAGCCACCCGAGATTGAGCA | TAGTAGCGACGGGCGGTGTG |
| SD-TNF-α | GGAGGGAGAACAGCAACTCC | GCCAGTGTATGAGAGGGACG |
| Mus-TNF-α | CAGCCGATGGGTTGTACCTT | GGCAGCCTTGTCCCTTGA |
| SD-IL-1β | TTGAGTCTGCACAGTTCCCC | TCCTGGGGAAGGCATTAGGA |
| Mus-IL-1β | GAAACCATGGCACATTCTGTTC | AATAGGTAAGTGGTTGCCCATCA |
| SD-IL-6 | AGAGACTTCCAGCCAGTTGC | AGTCTCCTCTCCGGACTTGT |
| Mus-IL-6 | AGAGGATACCACTCCCAACA | CAGTTTGGTAGCATCCATCA |
| SD-KLF14 | CGCATCCAAGCGACATCAGT | TGGGTACGCTGGTGTGACT |
| Mus-KLF14 | CTCCTGTGATTGGCTCGACTG | CGCTCCGAGAGAACTGCTTG |
